# IRF-1 Inhibits Angiogenic Activity of HPV16 E6 Oncoprotein in Cervical Cancer

**DOI:** 10.3390/ijms21207622

**Published:** 2020-10-15

**Authors:** Seung Bae Rho, Seung-Hoon Lee, Hyun-Jung Byun, Boh-Ram Kim, Chang Hoon Lee

**Affiliations:** 1 Division of Translational Science, Research Institute, National Cancer Center, Goyang, Gyeonggido 411-769, Korea; sbrho@ncc.re.kr; 2 Department of Life Science, Yong In University, Yongin, Gyeonggido 449-714, Korea; shlee@yongin.ac.kr; 3 Phamaceutical Biochemistry, College of Pharmacy and Integrated Research Institute for Drug, Dongguk University, Goyang 100-715, Korea; bhj1052@gmail.com

**Keywords:** human papillomavirus16 E6, IRF-1 tumour suppressor, protein-protein interaction, angiogenic activity, luciferase activity, VEGFR-2/PI3K/Akt signalling

## Abstract

HPV16 E6 oncoprotein is a member of the human papillomavirus (HPV) family that contributes to enhanced cellular proliferation and risk of cervical cancer progression via viral infection. In this study, interferon regulatory factor-1 (IRF-1) regulates cell growth inhibition and transcription factors in immune response, and acts as an HPV16 E6-binding cellular molecule. Over-expression of HPV16 E6 elevated cell growth by attenuating IRF-1-induced apoptosis and repressing p21 and p53 expression, but activating cyclin D1 and nuclear factor kappa B (NF-κB) expression. The promoter activities of p21 and p53 were suppressed, whereas NF-κB activities were increased by HPV16 E6. Additionally, the cell viability of HPV16 E6 was diminished by IRF-1 in a dose-dependent manner. We found that HPV16 E6 activated vascular endothelial growth factor (VEGF)-induced endothelial cell migration and proliferation as well as phosphorylation of VEGFR-2 via direct interaction in vitro. HPV16 E6 exhibited potent pro-angiogenic activity and clearly enhanced the levels of hypoxia-inducible factor-1α (HIF-1α). By contrast, the loss of function of HPV16 E6 by siRNA-mediated knockdown inhibited the cellular events. These data provide direct evidence that HPV16 E6 facilitates tumour growth and angiogenesis. HPV16 E6 also activates the PI3K/mTOR signalling cascades, and IRF-1 suppresses HPV16 E6-induced tumourigenesis and angiogenesis. Collectively, these findings suggest a biological mechanism underlying the HPV16 E6-related activity in cervical tumourigenesis.

## 1. Introduction

Worldwide, cervical cancer is the fourth most common cancer among women and is caused by infection with high-risk human papillomavirus (HPV). In 2018, 570,000 new cases of cervical cancer were recorded, with over 310,000 deaths [1,2]. More than 200 HPV genotypes have been identified in humans and animals. HPVs are clinically categorized into “low-risk” and “high-risk” groups. High-risk groups, such as those involving HPV16 and 18, are characterised by intraepithelial neoplasia and tumour progression [3,4].

HPV16 and HPV18 are the most widespread types, found approximately 80% of the cervical cancer cases worldwide. Particularly, high-risk transformation ability is mostly due to the up-regulation of the E6 and E7 viral oncoproteins, which associate with various cellular proteins, thus affecting their biological functions [4,5]. Generally, infection with high-risk HPV has been recognized as a crucial component for the development of cervical cancer. Moreover, the interaction of HPV E6 with various signalling cascades is related to cancer progression and metastasis. Physiologically, HPV infection plays a pivotal role in cervical carcinogenesis and is mostly disseminated via sexual or skin-to-skin contact. HPV-related tumourigenesis is mediated via enhanced expression of HPV E6 and E7 oncoproteins [6], which induce initial changes in epithelial cells, and inactivate the expression of tumour suppressor p53 and retinoblastoma (Rb) genes, respectively [7]. HPV E6 mediates various biological events underlying malignant transformation, such as regulation of transcription cell cycle-associated genes, enhanced ubiquitin-mediated degradation of p53 to suppress apoptotic cell death, control of cell shape and polarity, regulation of DNA repair, induction of telomerase activity, regulation of immune response, and cell communication [8,9]. Previously, studies have reported interaction with various different cellular components. For example, the interaction between E6 and p53 is mediated by ubiquitin ligase known as E6-associated protein (E6AP) [10,11], which reduces the down-stream activation of tumour necrosis factor receptor type 1-associated death domain protein (TRADD) by interfering with extrinsic apoptosis through cellular interaction with tumour necrosis factor receptor 1 (TNFR-1) [12]. In addition, E6 protein of the high-risk HPV binds to Fas-associated protein with death domain (FADD) facilitating its degradation and thereby interrupting the death-inducing signalling complex (DISC) formation and caspase-8 cleavage, and also directly interacts with caspase-8 targeted for proteasomal degradation [13,14,15,16,17].

Interferon regulatory factor-1 (IRF-1) is one of the members of IRF family. IRF-1 is a transcription factor involved in immune response, cell cycle regulation, induction of apoptotic cell death, post-translational modification, DNA damage, and suppression of oncogenesis [18,19,20,21,22]. In human haematological malignancies and solid cancers, IRF-1 is inactivated to interrupt apoptotic cell death and cell cycle arrest via genetic alteration, such as loss of heterozygosity (LOH) of gene and exon deletion including acute myelogenous leukaemia (AML), gastric cancer, oesopharyngeal cancer, breast cancer, renal cell carcinoma and stomach cancer [23,24,25,26,27,28,29,30].

IRF-1 binds to various cellular proteins, including nuclear factor kappa B (NF-κB), PUMA (p53 upregulated modulator of apoptosis), and XAF1 (XIAP-associated factor 1), which play a role in inflammatory response and tumourigenesis [31,32,33,34]. In addition, IRF-1 controls both SUMOylation and ubiquitination in tumour progression [22,35,36,37]. IRF family members share significant homology with the N-terminal 124 amino acids, which constitute a DNA-binding domain (DBD) characterised by the helix-loop-helix region of five tryptophan repeats [19,21,38,39,40]. The DBD is also required for homo-dimerization and nuclear location signal (NLS) [41]. The C-terminus of IRF-1 comprises a transactivation domain (TA) involving amino acids 185 and 256; however, this amino acid motif is not detected in the transcriptional repressor IRF-2. IRF-2 is the functional antagonist of IRF-1 [19]. It acts as an anti-angiogenic factor as well as tumour suppressor and oncoprotein [42,43,44].

Therefore, the precise physiological role of HPV16 E6 in cervical cancer metastasis and tumourigenesis remains largely unknown. To further explore the function of HPV16 E6 oncoprotein, we first used a yeast two-hybrid cDNA library with HPV16 E6 bait, and identified IRF-1 as a novel binding partner of HPV16 E6. We also investigated the ongoing biological role of PI3K/Akt/mTOR signalling pathway in the phosphorylation of vascular endothelial growth factor receptor 2 (VEGFR-2), or vascular endothelial growth factor (VEGF) and hypoxia-inducible factor-1α (HIF-1α) expression, and angiogenesis induced in vitro by HPV16 E6 oncoprotein in human umbilical vein endothelial cells (HUVECs) and cervical cancer cells. Formation of the cellular complex between HPV16 E6 and IRF-1 plays an important role in regulating the anti-tumour and anti-angiogenic mechanisms. Therefore, this molecular signalling mechanism has a significant role in new drug development.

## 2. Results

### 2.1. HPV16 E6 Oncoprotein Was Strongly Expressed by Inhibiting Apoptosis and Inactivating Caspase-3 in Human Cervical Cancer Cells

**Figure 1:**
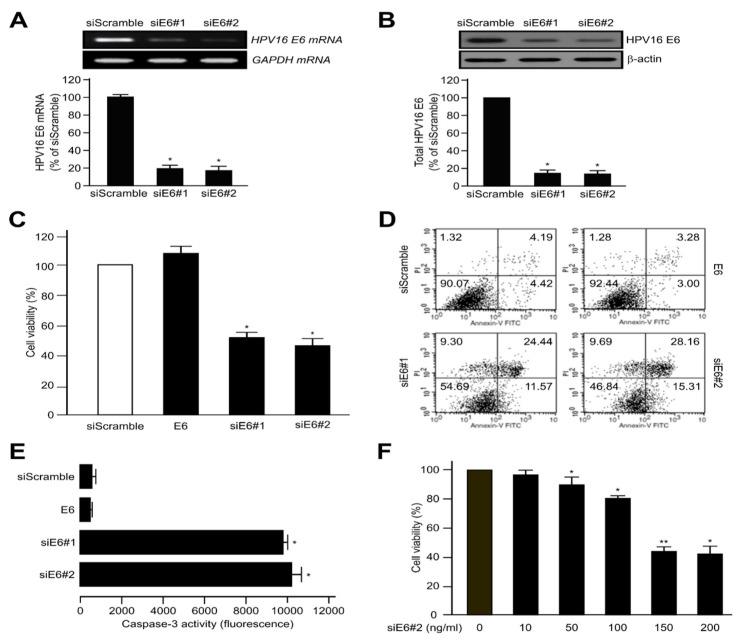
Human papillomavirus (HPV)16 E6 oncoprotein highly expressed by inactivating apoptosis in human cervical cancer cells. The control represents transfection of the siScramble. (**A**,**B**) Elimination of HPV16 E6 mRNA and protein expression by HPV16 E6-siRNA (siE6#1 or siE6#2) in CaSki cells. After transfection, the mRNA or protein expression of the cells was visualised via RT-PCR and western blots. The data are shown as the mean ± SD of at least three independent experiments. The mRNA and protein levels were evaluated via densitometric analysis and normalised to levels of the loading control; * *p* < 0.05, ** *p* < 0.01 compared with siScramble group. (**C**) The relative rates of cell proliferation were measured with CellTiter-Glo assay kit as described in the manufacturer’s protocols. Each data point shows triplicate samples, and the bars indicate the mean ± SD. * *p* < 0.05 , ** *p* < 0.01 compared with siScramble. (**D**) Early- and late-stage apoptosis induced by siScramble, HPV16 E6 (full-length), HPV16 E6-siRNA (siE6#1) or siE6#2 was determined using the fluorescein isothiocyanate (FITC)-labelled Annexin V assay. All experiments were repeated at least three times with similar results. (**E**) Caspase-3 activity was calculated using a microplate reader to measure the fluorescence based on the formula supplied by the manufacturer. (**F**) CaSki cells were transfected with various concentrations of the HPV16 E6-siRNA (siE6#2). Relative rates of cell proliferation were estimated as described in the supplier’s instructions using the CellTiter-Glo assay system. Results show the mean ± SD of at least three independent experiments carried out in triplicate. * *p* < 0.05, ** *p* < 0.01 compared with siScramble (0 ng/mL)).

We first transfected CaSki human cervical tumour cells with HPV16 E6 (siScramble) or HPV16 E6-siRNA (siE6#1 and siE6#2) in an effort to determine the role of HPV16 E6 in tumourigenesis. As shown in Figure 1A,B, the levels of mRNA and protein expression of HPV16 E6 in both HPV16 E6-siRNA (siE6#1 and siE6#2) were dramatically decreased compared with those of HPV16 E6 (siScramble). Next, we determined cell viability with and without HPV16 E6-siRNA. Controls were transfected with the siScramble. Based on the diminished number of cells observed, HPV16 E6-siRNA (siE6#1 and siE6#2)-transfected cell viability was less than that of siScramble cells by approximately 42% and 52%, respectively (Figure 1C). Subsequently, we used flow cytometry to confirm that the loss of viability was due to apoptosis. Cells were transfected with siScramble, HPV16 E6, siE6#1 or siE6#2, respectively. As expected, CaSki apoptotic cell death was higher in siE6#2 and siE6#1-expressing cells compared with siScrambles (Figure 1D). In addition, caspase-3 activity was conspicuously facilitated by transient transfection of siE6#2 or siE6#1 compared with siScramble and HPV16 E6 groups in CaSki cells (Figure 1E). Since siE6#2 was more effective than siE6#1, it was used for all experiments in the future. As a result, the transfection of siE6#2 (0~200 ng) induced apoptotic cell death in a dose-dependent manner (Figure 1F). Importantly, HPV16 E6 silencing increased the apoptosis of CaSki cells, indicating that HPV16 E6 negatively regulates apoptotic activity of cervical tumour cells.

### 2.2. HPV16 E6 Facilitates VEGF-Induced Endothelial Cell Migration, Proliferation, and Tube Formation In Vitro

**Figure 2:**
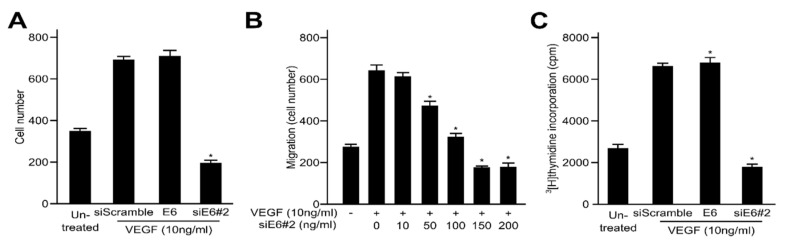

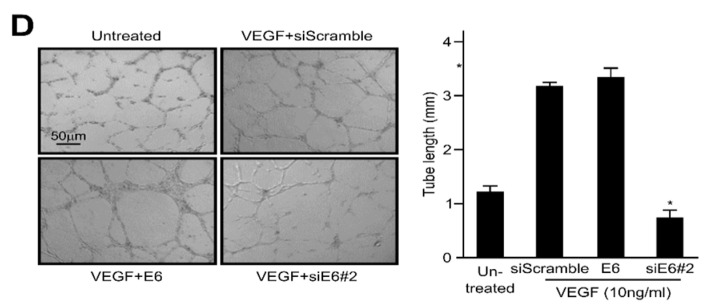
HPV16 E6 facilitates vascular endothelial growth factor (VEGF)-induced endothelial cell migration, proliferation and tubular network formation in vitro. (**A**,**B**) Boyden chamber-based migration assays were conducted to determine whether the transfected HPV16 E6 regulates the effects of VEGF on endothelial cell migration. After treatment with VEGF, HUVECs were transfected with siScramble, HPV16 E6 or HPV16 E6-siRNA (siE6#2) (**A**), or siE6#2 constructs at various concentrations (**B**). Human umbilical vein endothelial cells (HUVECs) were added to the top chamber of the Boyden Transwell chamber (pore size, 8 μm), fixed, and then stained with Haematoxylin and Eosin (H&E). The number of migrated cells was evaluated under a light microscope. Three independent experiments were conducted in triplicate; * *p* < 0.05 compared with siScramble. (**C**) Effects of HPV16 E6 on endothelial cell proliferation. HUVECs were grown and incubated for 3 days with or without VEGF. The c.p.m. of [^3^H]thymidine was calculated using a liquid scintillation counter. Each data point represents triplicate samples, and the bars indicate the mean ± SD; * *p* < 0.05 versus siScramble. All experiments were performed at least three times with consistent and similar results. (**D**) HUVECs were transfected with siScramble, HPV16 E6 or HPV16 E6-siRNA (siE6#2) and then grown on growth factor-reduced Matrigel in the presence or absence of 10 ng/mL VEGF. The capillary-like tubular network formation was monitored under an inverted microscope; scale bars represent 50 μm. The tube lengths were quantified and expressed as mean ± SD; * *p* < 0.05 versus siScramble. Each data bar represents the mean ± SD of three independent experiments that yielded similar values.

VEGF is one of the key regulators of angiogenesis in the pathophysiology of solid tumours. Therefore, the inhibition of VEGF expression has been shown to repress tumour growth including cell migration, invasion and metastasis. To determine whether HPV16 E6 controls the effects of VEGF on cell proliferation in HUVECs, angiogenesis was evaluated based on cell migration, proliferation and formation of capillary-like tubular structure in the endothelial cells. We initially investigated the HPV16 E6-specific regulation of VEGF-induced migration of endothelial cells utilizing Transwell migration assays. As expected, VEGF accelerated the migration of the untransfected cells and the siScramble-transfected cells compared with that of uninduced cells. Our results indicate that HPV16 E6 ectopic expression significantly elevated VEGF-induced cell migration, whereas HPV16 E6-siRNA did not (Figure 2A). We conducted additional experiments using a series of HPV16 E6 (siE6#2) concentrations via transient transfection. As seen in Figure 2B, VEGF enhanced cell migration of empty siE6#2-transfected HUVECs when compared with that of uninduced cells, as expected. The elimination of the over-expressed HPV16 E6 by siE6-siRNA remarkably diminished the stimulatory effects of VEGF on the migration of HUVECs. The effects of HPV16 E6 on VEGF-induced proliferation of endothelial cells were assessed via [^3^H]thymidine incorporation analysis. HPV16 E6 elevated VEGF-induced DNA synthesis of HUVECs, while siE6-siRNA significantly suppressed it (Figure 2C). Therefore, over-expressed HPV16 E6 strongly accelerates key events in the angiogenic process induced by VEGF, such as cell migration, invasion and proliferation of endothelial cells in vitro. Subsequently, we demonstrated the angiogenic effects of HPV16 E6 on VEGF-induced formation of capillary-like tubular structure on Matrigel using an in vitro angiogenesis model in HUVECs. As shown in Figure 2D, untreated or siScramble cells incubated with VEGF formed a capillary-like tubular structure on Matrigel. By contrast, the ectopic expression of HPV16 E6-siRNA (siE6#2) completely disrupted the formation of VEGF-induced tubular structure. The inhibitory effect of siE6#2 on VEGF-induced tube formation was completely reversed by HPV16 E6 transient transfection. These findings strongly indicate that HPV16 E6 specifically controls VEGF-induced tube formation in HUVECs.

### 2.3. HPV16 E6 Directly Binds with VEGFR-2 but Not VEGFR-1

**Figure 3:**
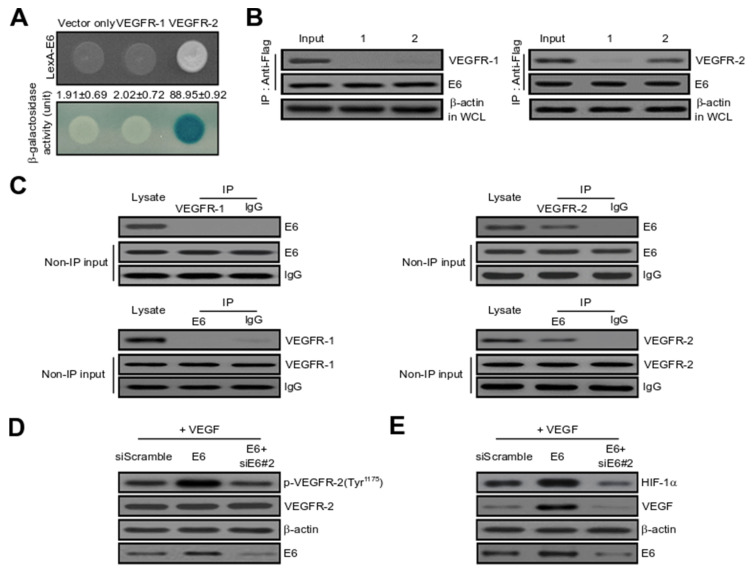

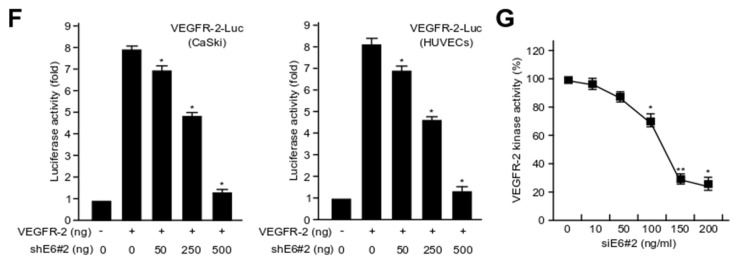
HPV16 E6 directly interacts with VEGFR-2, but not VEGFR-1. (**A**) Positive interactions were validated by determining the cell growth on a medium lacking leucine (upper panel) and by the formation of blue colonies (lower panel). β-galactosidase activity (unit), calculated by adding *_O_*-nitrophenyl β-_D_-galactopyranoside (ONPG) reagent, is shown below the corresponding lanes. All experiments were repeated at least three times with similar results. (**B**) Co-immunoprecipitation (Co-IP) of HPV16 E6 with VEGFR-1 or VEGFR-2. Immunoprecipitation (IP) of transfected cells was conducted using anti-Flag antibodies in total lysates, followed by immunoblotting with anti-HPV16 E6, anti-VEGFR-1 and anti-VEGFR-2 antibodies; (left panel) lane 1, pcDNA3.1 (empty-inserted expression vector only) and pcDNA3.1/Flag-HPV16 E6 transfectant; lane 2, pcDNA3.1/Flag-HPV16 E6, and pcDNA3.1/VEGFR-1 transfectant; (right panel) lane 1, pcDNA3.1 (empty expression vector only) and pcDNA3.1/Flag-HPV16 E6 transfectant; lane 2, pcDNA3.1/Flag-HPV16 E6 and pcDNA3.1/VEGFR-2 transfectant. (**C**) Endogenous proteins in whole cell lysates of human embryonic kidney 293T (HEK293T) cells were introduced into Co-IP with an antibody as indicated, followed by immunoblotting with an anti-HPV16 E6 or anti-VEGFR-2 antibody. Rabbit immunoglobulin G (IgG) and VEGFR-1 served as an IP-negative control. Input (non-IP) immunoblotting data indicated the integrity of cell lysates used for IP. All experiments were repeated at least three times with similar results. (**D**) HUVECs were treated with VEGF and then control-transfected or transfected with HPV16 E6 or HPV16 E6 plus HPV16 E6-siRNA (siE6#2). Phosphorylation of VEGFR-2 (Tyr-1175) was demonstrated using the specific antibody. The VEGFR-2 band ensured equal loading of samples. All experiments were conducted at least three times with consistent and similar results. (**E**) Cells were incubated with 10 ng/mL VEGF, and then incubated with either siScramble-transfected or transfected with HPV16 E6 or HPV16 E6 plus HPV16 E6-siRNA (siE6#2). Expression levels of VEGF and HIF-1α protein were determined via immunoblotting analysis. All experiments were repeated at least three times with similar results. (**F**) Inhibitory effect of VEGFR-2-dependent transcription by HPV16 E6-siRNA (siE6#2). CaSki cervical cells and HUVECs were co-transfected with 500 ng of VEGFR-2-Luc, 500 ng of a VEGFR-2 expression plasmid (pcDNA3.1/VEGFR-2) and increasing concentrations of plasmid-encoding shE6#2 (pcDNA3.1/Flag-siE6#2) (50, 250 and 500 ng). Experiments were conducted in triplicate, and error bars show the mean ± SD; * *p* < 0.05 compared with control. (**G**) Suppression of VEGFR-2 kinase activity of HPV16 E6-siRNA (siE6#2) was analysed using an in vitro HTScan VEGFR-2 kinase assay kit combined with colourimetric detection according to the supplier’s instructions. Data are expressed as mean ± SD from three independent experiments; * *p* < 0.05; ** *p* < 0.01 compared with zero concentration.

To elucidate the cellular mechanism of HPV16 E6 in tumour angiogenesis, we used yeast two-hybrid (Y2H) and co-immunoprecipitation (Co-IP) assays. We initially investigated the intracellular binding of HPV16 E6 with VEGFR-1 and VEGFR-2. As shown in Figure 3A, β-galactosidase was completely activated (88.95 ± 0.92) in the protein-protein interaction (PPI) between HPV E6 and VEGFR-2 but not between HPV16 E6 and empty expression vector (vector only: 1.91 ± 0.69) or VEGFR-1 (2.02 ± 0.72). Therefore, VEGFR-1 was utilized as a negative control in subsequent experiments. We next used Co-IP to ascertain the direct interaction between HPV16 E6 and VEGFR-2 in vivo. DNA constructs expressing HPV16 E6 (pcDNA3.1/Flag-HPV16 E6) and VEGFR-1 or VEGFR-2 (pcDNA3.1/VEGFR-1 or VEGFR-2) or pcDNA3.1/Flag-HPV16 E6 and vector alone (pcDNA3.1) were co-transfected into cells. Immunoprecipitation of whole cell lysates from transfected cells was performed utilizing an anti-Flag antibody, and the precipitated 20 µg proteins were immunoblotted with anti-HPV E6, anti-VEGFR-1 or anti-VEGFR-2 antibodies. As seen in Figure 3B, pcDNA3.1/VEGFR-2 co-immunoprecipitated with pcDNA3.1/Flag-HPV16 E6 (lane 2 in the upper right panel), but not with pcDNA3.1 (vector only) or VEGFR-1 (lane 1 in the upper left panel). We demonstrated the interaction between endogenous HPV16 E6 and VEGFR-2. The oncoprotein HPV16 E6 directly bound with VEGFR-2 (right panel) but not VEGFR-1 (left panel) (Figure 3C). VEGFR-2 is an essential regulator of VEGF-induced endothelial cell function. Therefore, the angiogenic effect of HPV16 E6 on VEGF-induced VEGFR-2 phosphorylation was observed in vitro in HUVECs. As seen in Figure 3D, the ectopic expression of HPV16 E6 significantly elevated VEGF-induced VEGFR-2 phosphorylation (Tyr-1175). In contrast, overexpression of HPV16 E6-siRNA (siE6#2) dramatically reduced HPV16 E6-induced VEGFR-2 phosphorylation. These new findings suggest that HPV16 E6 significantly enhanced VEGFR-2 phosphorylation in vitro in HUVECs. In general, the expression levels of HIF-1α or VEGF protein play a critical role in PI3K/Akt signalling. In rapidly growing cancers, hypoxic conditions greatly activate the expression of HIF-1α transcription factor, which in turn triggers VEGF protein expression in tumour cells. Subsequently, VEGF expression controls other angiogenic components and therefore plays an essential role in the regulation of tumour angiogenesis. To inhibit the formation of new blood vessels in cancer, it is important to suppress the expression of VEGF or HIF-1α proteins in tumour cells. As shown in Figure 3E, over-expression of HPV16 E6 remarkably accelerated the expression of VEGF-induced HIF-1α or VEGF protein, whereas the activation of VEGF-induced protein expression by HPV16 E6 was completely suppressed by transient transfection with HPV16 E6-siRNA. These results suggest that HPV16 E6 significantly facilitated the expression of HIF-1α or VEGF protein in HUVECs in vitro. Subsequently, to demonstrate whether HPV16 E6 gene silencing (shE6#2) regulates VEGFR-2, the effect of shE6#2 on VEGFR-2 transcription activity was estimated with a luciferase reporter-gene analysis system, which used a construct carrying a VEGFR-2 promoter fused to the luciferase gene. Luciferase activity was progressively reduced by transient transfection of shE6#2 in a dose-dependent manner in CaSki and HUVECs (Figure 3F). Importantly, the decline in transcriptional activity was also evident in SiHa cervical and MCF-7 breast cancer cells (data not shown). Thus, these data further underscore the role of HPV16 E6-siRNA (siE6#2) in regulating VEGFR-2 activity. We next determined whether HPV16 E6-siRNA (siE6#2) diminished phospho-VEGFR-2 levels via suppression of kinase activity by demonstrating the effects of HPV16 E6-siRNA (siE6#2) on VEGF-triggered phospho-VEGFR-2 using ELISA. The data confirmed that HPV16 E6-siRNA (siE6#2) attenuated VEGFR-2 kinase activity in a dose-dependent manner (Figure 3G).

### 2.4. HPV16 E6 Directly Interacts with IRF-1

**Figure 4:**
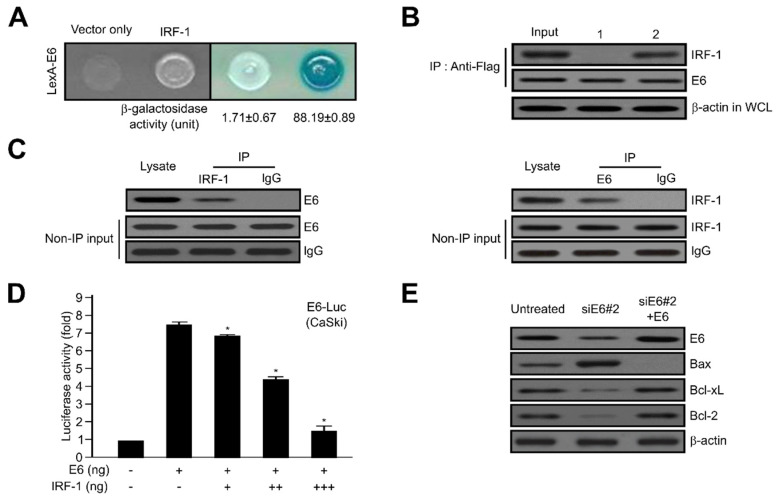
Direct interaction between HPV16 E6 and interferon regulatory factor-1 (IRF-1) tumour suppressor proteins. (**A**) Positive interactions were monitored to determine the cell growth in the medium lacking leucine and β-galactosidase activity (unit) using *o*-nitrophenyl β-D-galactopyranoside (ONPG). (**B**) Co-immunoprecipitation of HPV16 E6 with IRF-1. Immunoprecipitation was conducted using an anti-Flag antibody and whole cell lysates from transfected cervical cancer cells. After immunoprecipitation, the precipitated proteins were detected using anti-HPV16 E6 and anti-IRF-1 antibodies. Lane 1, pcDNA3.1 (vector only) and pcDNA3.1/Flag-HPV16 E6 transfectants; lane 2, pcDNA3.1-IRF-1 and pcDNA3.1/Flag-HPV16 E6 transfectants. β-actin served as a loading control. (**C**) Co-immunoprecipitation of endogenous HPV16 E6 and IRF-1 indicated an interaction between HEK293T cells. Rabbit immunoglobulin G (IgG) served as an IP-negative control. (**D**) Activity of HPV16 E6-dependent transcription by IRF-1 tumour suppressor. CaSki cervical tumour cells were co-transfected with 500 ng of HPV16 E6-Luc, 500 ng of a HPV16 E6 expression plasmid (pcDNA3.1/HPV16 E6), and increasing concentrations of plasmid-encoding IRF-1 (pcDNA3.1/Flag-IRF-1, IRF-1) (50, 250, and 500 ng). Each data bar represents the mean ± SD of four independent experiments that yielded similar results; * *p* < 0.05 compared with control. (**E**) Expression levels of Bcl-2 family genes were determined via western blotting analysis of cervical cancer cells transfected with HPV16 E6 or HPV16 E6-siRNA. β-actin served as a protein loading control, and antibodies against HPV16 E6, Bax, Bcl-xL, and Bcl-2 were used.

To explore the detailed cellular function of HPV16 E6 oncoprotein, we utilized Y2H screening and co-immunoprecipitation (Co-IP) assay system. In vivo, the positive interaction was determined via both cell growth and *_O_*-nitrophenyl β-_D_-galactopyranoside (ONPG) β-galactosidase activity. An empty-inserted expression vector (vector only) was used as the negative control. As shown in Figure 4A, the activity of β-galactosidase activity for protein binding between HPV16 E6 and IRF-1 was total (88.19 ± 0.89), but not with empty vector (vector only: 1.71 ± 0.67). To further verify the direct binding between HPV16 E6 and IRF-1 observed in the Y2H assay, the binding strength was further validated via Co-IP. DNA constructs expressing HPV16 E6 (pcDNA3.1/Flag-HPV16 E6) and IRF-1 (pcDNA3.1/IRF-1) or pcDNA3.1/Flag-HPV16 E6 and vector alone (pcDNA3.1) were co-transfected into human embryonic kidney 293T (HEK293T) cells. Immunoprecipitation was then performed in lysates derived from transfected cells using an anti-Flag antibody, and the precipitated proteins were developed with anti-HPV16 E6 or anti-IRF-1 antibodies. As seen in Figure 4B, pcDNA3.1/IRF-1 protein co-immunoprecipitated with pcDNA3.1/Flag-HPV16 E6 (lane 2), but not with pcDNA3.1 (vector only) (lane 1). Continuously, we ascertained the protein binding between endogenous HPV16 E6 and IRF-1. The oncoprotein HPV16 E6 directly binds with IRF-1 tumour suppressor, but not IgG used as a negative control (Figure 4C).

Subsequently, to elucidate whether IRF-1 controls HPV16 E6, the effect of IRF-1 on HPV16 E6 transcription activity was calculated using a luciferase reporter-gene assay system, which was based on a construct carrying an HPV16 E6 promoter fused with the luciferase gene. Luciferase activity was progressively diminished via transient transfection of tumour suppressor IRF-1 in a dose-dependent manner in CaSki cancer cells (Figure 4D). Thus, these data further elucidate the importance of IRF-1 as a control for HPV16 E6 activity. We next demonstrated HPV16 E6-triggered cell growth mediated by the expression of apoptosis-regulatory proteins, such as Bax, Bcl-xL, and Bcl-2. The indicated proteins are well-known major components of apoptosis and cell growth. As shown in Figure 4E, Bax expression was diminished, whereas the expression of Bcl-xL and Bcl-2 was enhanced by HPV16 E6, compared with non-transfectants. To further validate these results, cells transfected with a specific HPV16 E6 siRNA (siE6#2) showed elevated Bax expression, while the expression of Bcl-xL and Bcl-2 was significantly diminished by siE6#2-RNA.

### 2.5. IRF-1 Controls the Expression of Cell Cycle- and Apoptosis-Regulatory Proteins

Regulation of the cell cycle plays a pivotal role in cellular homeostasis. It entails DNA replication and repair as well as inhibition of uncontrolled cell division. This functional mechanism is generally controlled by two major classes of regulatory molecules, cyclin family and cyclin-dependent kinases (CDKs) [45]. In particular, CDKs, which are also known as serine/threonine kinases, play a vital role in the complex feedback regulation of cell cycle progression.

**Figure 5:**
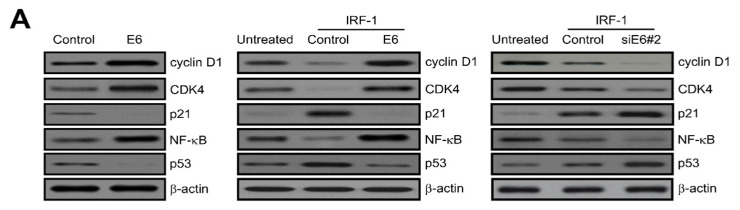

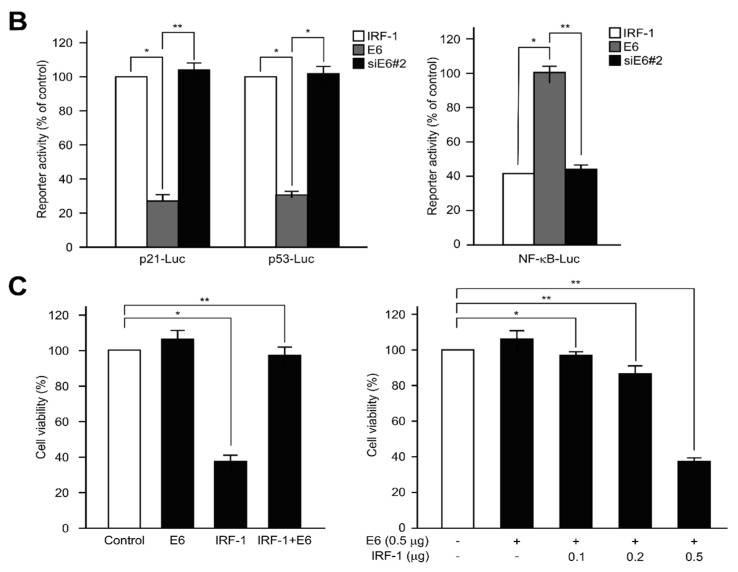
Effects of HPV16 E6 on IRF-1-induced apoptosis, p53, and nuclear factor kappa B (NF-κB) activity. (**A**) CaSki cells were transfected with HPV16 E6 expression plasmid (pcDNA3.1/HPV16 E6) (left panel) and IRF-1 for 24 h, and IRF-1-expressing cells were then transiently transfected with a control, HPV16 E6 (middle panel) or siE6#2 (right panel), respectively. After 48 h, cells were harvested and treated with lysis buffer. Whole cell lysates were subsequently subjected to western blotting analysis. Protein expression of cell cycle and apoptosis-related genes was observed. Protein expression was visualized through immunoblotting using specific antibodies. All experiments were repeated at least three times with similar results. (**B**) Promoter activities of p21, p53, and NF-κB were estimated via luciferase reporter-gene analysis with a p21 promoter reporter (p21-Luc), p53 (p53-Luc) and NF-κB (NF-κB-Luc), respectively. For instance, the p21-luciferase reporter gene constructs or promoter-less plasmid (vector only) were transfected into IRF-1-induced cervical tumour cells. Briefly, cells were incubated for 24 h and then added to the lysis buffer. After centrifugation, whole cells were lysed and mixed with luciferase reaction substrate to measure the luciferase activity. All results shown are representative of three independent experiments, and the bars indicate the mean ± SD. * *p* < 0.05; ** *p* < 0.01 compared with control group. (**C**) CaSki cells were transfected with HPV16 E6, IRF-1, or HPV16 E6 plus IRF-1, respectively. Relative rates of cell proliferation were evaluated as described in the manufacturer’s instructions provided for the CellTiter-Glo assay system. Results represent the mean ± SD of at least three independent experiments conducted in triplicate; * *p* < 0.05; ** *p* < 0.01 compared with control.

To investigate the effect of IRF-1 found as an E6 binding protein on the E6-induced proliferation, we examined the expression of cell cycle- and apoptotic cell death-regulatory proteins via western blotting analysis. As shown in Figure 5A (left panel), the ectopic expression of HPV16 E6 considerably increased the levels of cyclin D1, CDK4 and nuclear factor kappa B (NF-κB), but diminished the expression of p53 or p21, acting as a CDK inhibitor. These results establish that the ectopic expression of HPV16 E6 strongly inhibits IRF-1-induced apoptosis (Figure 5A, middle panel). NF-κB and p53 tumour suppressor proteins are major components of cell growth and apoptosis. To elucidate the biological effects of HPV16 E6 on IRF-1-induced apoptotic cell death, cells were transfected with either an HPV16 E6 expression vector or siE6#2, and the promoter activity was then measured via dual luciferase reporter gene analysis. As shown in Figure 5B, the promoter activities of p21 and p53 were remarkably diminished, while NF-κB activity was significantly elevated by HPV16 E6 over-expression. Taken together, these results clearly demonstrate that HPV16 E6 controls apoptosis regulator protein levels via reduction of p53 and acceleration of NF-κB activity during IRF-1-induced apoptosis in cervical tumour cells. We also investigated how IRF-1 tumour suppressor regulates HPV16 E6-induced cell proliferation. CaSki cervical cancer cells were transfected with control, HPV16 E6, IRF-1 or IRF-1 plus HPV16 E6, respectively. Control transfectant involved the empty-inserted expression vector only. According to the diminished number of cells after 24 days, which indicates cell viability, IRF-1-transfected cells were significantly reduced to 55~60% compared to control, but elevated in HPV16 E6-transfected cells. Subsequently, to elucidate the cellular biological function of HPV E6 during IRF-1-induced apoptosis, IRF-1-induced apoptosis cells were transfected with HPV16 E6. As shown in Figure 5C (left panel), HPV16 E6 enhances cancer cell apoptosis induced by IRF-1. To confirm these results, HPV16 E6-overexpressing stable cells (0.5 μg) were transfected with various concentration of IRF-1 (0~0.5 μg). As a result, the apoptotic cell death was progressively elevated in a dose-dependent manner (Figure 5C, right panel).

### 2.6. HPV16 E6 Activates Major Component Phosphorylation in PI3K/mTOR Signalling Pathway

**Figure 6:**
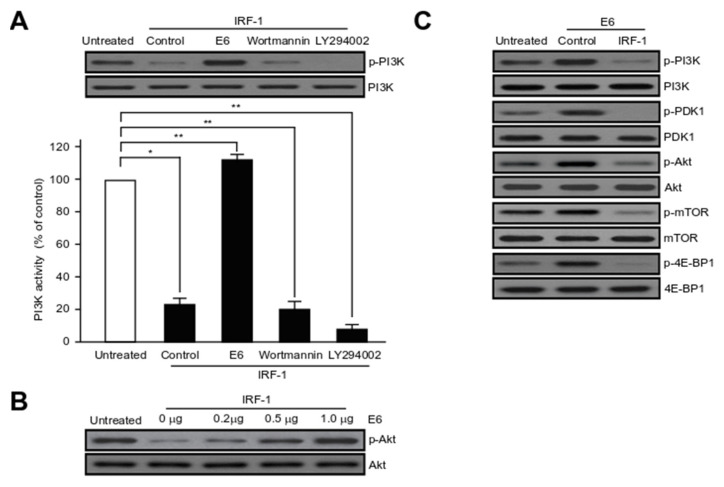
HPV16 E6 oncoprotein enhances PI3K/mTOR phosphorylation. (**A**) The effect of HPV16 E6 on PI3K activity in the presence of IRF-1 was evaluated using an in vitro PI3 kinase assay system. As shown in the graph, the transfected cells with IRF-1 were transfected or treated with the control, HPV16 E6 expression plasmid, Wortmannin or LY294002 for 24 h, and harvested and immunoblotted to measure the indicated levels of protein expression. PI3K served as a protein loading control. All experiments were repeated at least three times with similar results. Protein bands were evaluated via densitometric analysis and normalized to levels of the loading control; * *p* < 0.05; ** *p* < 0.01 compared with control. (**B**) IRF-1-expressing cells were transfected with various concentrations of the HPV16 E6 expression plasmid. Phosphorylation of Akt was detected by immunoblotting analysis. Protein band for non-phosphorylated Akt ensured equal loading of the samples. All experiments were performed at least three times with consistent and similar results. (**C**) Cells were transfected with the untreated, HPV16 E6 (control) or HPV16 E6 plus IRF-1. The total cells were harvested and treated with lysis buffer. After centrifugation, equal amounts of protein (20~25 μg) were separated on 8~12% SDS-PAGE, followed by immunoblotting analysis with specific antibodies (PI3K/phosphorylated PI3K, PDK1/phosphorylated PDK1, Akt/phosphorylated Akt, mTOR/phosphorylated mTOR, and 4E-BP1/phosphorylated 4E-BP1). All experiments were conducted at least three times with consistent and similar results. Non-phosphorylated form (indicated PI3K, PDK, Akt, mTOR, and 4E-BP1) and β-actin ensured equal loading of the samples.

The PI3K pathway is involved in the HPV-induced proliferation, survival, and angiogenesis of cancer cells [46,47]. Especially the PI3K pathway is unique, in that all of the major players have been reported to be frequently mutated or amplified in HPV-induced cancers [48,49,50,51,52]. We have also known from previous studies that IRF-1 inhibits PI3K signalling in HUVEC [44]. To elucidate how E6 and its binding protein, IRF-1, affect the PI3K/AKT pathway in cervical cancer cells, we investigated the role of PDK1, Akt, mTOR, and 4E-BP1, which are downstream signalling regulators of PI3K. Whole cell lysates from IRF-1-expressing cells (control) and HPV16 E6-transfected cells were detected via western blotting analysis. HPV16 E6 enhanced the phosphorylation of PI3K, Akt, PDK1, and mTOR in HPV16 E6-induced cervical tumourigenesis, cell migration, invasion and metastasis. As shown in Figure 6A, IRF-1-induced PI3K and Akt de-phosphorylation were completely reversed by HPV16 E6 oncoprotein. Effects of IRF-1 was comparable to that of wortmannin and LY294002, which are well-known PI3K inhibitors. Further, the ectopic expression of HPV16 E6 progressively enhanced Akt phosphorylation in a dose-dependent manner (Figure 6B). Subsequently, we determined the phosphorylation of key components in the PI3K/mTOR signalling cascades. As shown in Figure 6C, HPV16 E6 significantly elevated PDK1 phosphorylation, as well as the phosphorylation of mTOR and 4E-BP1. Thus, we observed that IRF-1 over-expression remarkably suppression the expression of the PI3K/mTOR signalling pathway-related proteins. All these results strongly suggest that HPV16 E6 rescues IRF-1-triggered apoptosis by interfering with the PI3K/PDK1/mTOR signalling pathway in cervical tumour cells.

## 3. Discussion

HPV infection plays an important role in cervical carcinogenesis. The pathogenesis of HPV infection is mediated via over-expression of viral oncoproteins that suppress the expression of a variety of cellular proteins and interfere with the biological processes underlying cell cycle, cell proliferation and apoptotic cell death. The viral and host cellular transformation that trigger cervical carcinogenesis provide profound insight into the pathophysiology of the disease as well as motivate the development of molecular targeted therapy [7]. Especially, the etiology of cervical carcinogenesis is closely associated with high-risk subtypes of human papillomavirus (HPV), which involve the E6 and E7 oncoproteins that contribute to early changes in epithelial cells [53,54]. HPV E6 and E7 viral proteins play a key role in host cell immortalization and transformation by inactivating the p53 and retinoblastoma (pRb) tumour suppressor proteins, or by directly binding with various cellular response factors such as the inhibitor of growth 4 (ING4) [7,55,56]. Inactivation of these host tumour suppressor proteins interrupts the DNA repair and apoptotic cell death mechanism, leading to rapid cell proliferation. Generally, multiple proteins involved in DNA repair, cell proliferation, angiogenesis, as well as mitogenesis are highly expressed in cervical intraepithelial neoplasia (CIN) and tumour [7]. Recently, He et al. reported that the Hippo-Yap signalling pathway plays an important role in the development of cervical tumours [57]. In the Hippo signalling cascade, the HPV E6 directly binds with the Yes-associated protein (YAP1) to initiate and accelerate tumour progression in cervical oncogenesis.

The key findings of the present study are as follows: (a) ectopic expression of HPV16 E6 accelerates tumour cell growth by inhibiting caspase-3 activity in CaSki cells. (b) Induction of VEGFR-2 phosphorylation by HPV16 E6 influences the expression of HIF-1α and VEGF proteins via interaction with VEGFR-2 in cervical and HUVEC. (c) HPV16 E6 directly binds with IRF-1 and regulates the cell cycle of HPV16 E6 by IRF-1 tumour suppressor. (d) HPV16 E6 activates the phosphorylation of PI3K/mTOR signalling components resulting in angiogenesis of cervical tumour.

Caspase-3 activity was substantially enhanced by transient transfection to siE6#2 or siE6#1 compared with siScramble and HPV16 E6 group in CaSki cervical cancer cells (Figure 1E). Since siE6#2 was more effective than siE6#1, it was use for all further experiments. As a result, the transfection of siE6#2 (0~200 ng) led to a dose-dependent elevation in apoptosis (Figure 1F). These results were consistent with reports of Bousarghine et al. and Gu et al. [58,59]. These results suggest that our E6 siRNA works properly in cervical cancer cells and that E6 siRNA can be a therapeutic agent for E6 positive cervical cancer patients.

Tumour angiogenesis plays an important role in cancer growth and metastasis of endothelial cells mediated via a variety of cellular components, and VEGF and HIF-α represent powerful angiogenic regulators. The VEGFR family consists of three types of tyrosine kinase receptors known as VEGFR-1 (Flt-1; fms-like tyrosine kinase), VEGFR-2 (KDR/Flk-1; kinase-insert domain containing receptor) and VEGFR-3 (Flt-4), and VEGFR-2 is more critical in controlling mitosis and vascular permeability of endothelial cells [60,61,62,63,64]. Generally, the biological mechanism controlling VEGF expression is thought to be associated with hypoxic conditions. Hypoxic cancer cells exhibit rapid VEGF expression, which is directly linked to hypoxia-induced factor HIF-1α. HIF-1α plays an important role in the initiation and progression of tumour angiogenesis by controlling a variety of angiogenic elements [65,66]. Besides, it was also confirmed in Figure 2 that E6 siRNA inhibits the VEGF-induced proliferation and migration, tube formation of HUVECs and tube formation. These results were consistent with those of Zhang et al., although the conditions of experiments were partially different [67]. These results suggested that HPV16 E6 is involved in VEGF-induced endothelial cell migration, proliferation and capillary-like tubular network formation.

In Figure 3, we found that E6 binds to VEGFR2 and E6 is involved in phosphorylation of VEGFR-2 (Tyr-1175). To the best of our knowledge, it seems the first time we have shown that E6 binds directly to VEGFR2. In addition, HPV16 E6 manifested potent pro-angiogenic activity and facilitated HIF-1α and VEGF expression by acting as a major modulator of angiogenesis (Figure 3). As shown in Figure 3E, ectopic expression of HPV16 E6 significantly enhanced the expression of VEGF-induced HIF-1α or VEGF protein, while the effect of HPV16 E6 on VEGF-induced protein expression was completely abrogated by transient transfection with HPV16 E6-siRNA. These results indicate that HPV16 E6 dramatically accelerated the expression of HIF-1α or VEGF protein in vitro in HUVECs. In addition, HPV16 E6 gene silencing attenuated VEGFR2 expression and VEGFR-2 kinase activity in a dose-dependent manner (Figure 3F,G). In the future, it is considered very interesting to investigate how the direct interaction between E6 and VEGFR2 is involved in E6-induced angiogenesis. In addition, this connection of E6-VEGFR2 is thought to be worth checking whether it exists in other carcinomas involving E6.

As reported, HPV E6 activated cell viability by interfering with the biological functions of various cellular components via protein-protein interactions. A basic mechanism underlying HPV E6-induced cell proliferation involves E6-associated protein (E6AP), which triggers the degradation of p53 via ubiquitin-dependent proteolysis [68,69]. However, HPV E6 facilitates cell transformation without p53 degradation. The ING4-induced p53 acetylation combined with p53 was diminished by HPV E6 independent of p53 degradation [56,70]. Based on the direct interaction between HPV16 E6 and IRF-1, our results establish that HPV16 E6 regulates the levels of apoptosis mediator protein via attenuation of p53 and acceleration of NF-B activity during IRF-1-induced apoptotic cell death in cervical tumour cells (Figure 4 and Figure 5). Similarly, it has been reported that E7 directly binds to IRF-1 in NIH/3T3 cells [71]. Therefore, our results that E6 interacts with IRF-1, which is involved in the antiviral interferon pathway, might also serve as a new clue to explain how E6 can inhibit interferon in antiviral responses.

The PI3K/Akt signalling cascade plays an important role in the regulation of signal transduction, and mediates a variety of biological phenomena such as angiogenesis, cell proliferation, apoptosis and cell metabolism [72,73]. Notably, the PI3K pathway is unique, in that all of the major components of this pathway are frequently amplified or mutated in HPV-induced cancers [48,49,50,51,52]. We found that IRF-1 over-expression remarkably suppressed the expression of PI3K/mTOR signalling pathway-related proteins in cervical cancer cells (Figure 6). These experimental results are consistent with our previous experimental results that IRF-1 inhibits the PI3K/AKT pathway in HUVEC. However, this result is the first to suggest the possibility that a direct interaction between E6 and IRF1 will be involved in the activation of PI3K/AKT [44].

In previous studies, in vivo tumourigenicity was increased at IRF-1 deficient breast carcinoma cells [74]. Activation of IL-6/STAT3 signalling was observed in mice implanted with cervical cancer cells containing high-risk HPV E6 [75]. Walch-Rückheim et al. show that higher IRF-1 expressed-cervical cancer patients responded significantly better to radio/chemotherapy [76]. In addition, Lee et al. reported the existence of splicing variants of IRF-1 lacking the part responsible for the activity of the IRF-1 transcription factor in tissues of uterine cancer patients [29].

In summary, our findings identify for the first time that IRF-1 tumour suppressor as a novel HPV16 E6-interacting protein. This interaction is critical for HPV16 E6-mediated cell proliferation and angiogenic function by accelerating NF-B, VEGF and HIF-1α repression. In addition, the molecular mechanism underlying the inhibitory effect of HPV16 E6 may involve inactivation of VEGFR-2 phosphorylation, and suppression of upstream and downstream activation of the PI3K/mTOR signalling pathways. Collectively, all our data provide information that will improve understanding of the cellular and molecular mechanism for HPV16 E6 biological function, and importance of the IRF-1 tumour suppressor in controlling tumour growth and angiogenesis of HPV16 E6 oncoprotein in cervical tumourigenesis.

## 4. Materials and Methods

### 4.1. Cell Lines, Reagents, and Antibodies

Human cervical carcinoma cell lines (CaSki and SiHa) and human embryonic kidney 293T (HEK293T) cells were purchased from the American Type Culture Collection (ATCC, Manassas, VA, USA) and were maintained in monolayer cultures according to ATCC recommendations. HUVECs were purchased from Clonetics (Walkersville, MD, USA), and seeded on 0.3% gelatin-coated dishes (Sigma, St. Louis, MO, USA) using the EGM-2 BulletKit medium (Clonetics). Wortmannin and LY294002, which are PI3K inhibitors, were obtained from Sigma (St. Louis, MO, USA). The following antibodies were used in this study: anti-HPV16 E6 (ab226447), anti-IgG (Abcam, Cambridge, UK), anti-IRF-1, anti-Flag, anti-cyclin D1, anti-CDK4, anti-NF-κB (Santa Cruz Biotechnology, Santa Cruz, CA, USA), anti-Bax, anti-Bcl-xL, anti-Bcl-2, anti-p53, anti-VEGFR-1, anti-VEGFR-2, anti-phospho-VEGFR-2(Tyr-1175), anti-HIF-1α, anti-PI3K, anti-phospho-PI3K, anti-Akt, anti-phospho-Akt(Ser473), anti-PDK-1, anti-phospho-PDK-1(Ser241), anti-mTOR, anti-phospho-mTOR(Ser2448), anti-4E-BP1, anti-phospho-4E-BP1(Thr70) (Cell Signalling, Beverly, MA, USA), anti-p21, anti-VEGF (Ab-1; Oncogene, Cambridge, MA, USA), and anti-β-actin (Sigma, St. Louis, MO, USA).

### 4.2. Flow Cytometric Analysis of Apoptosis and Cell Viability

Cell proliferation was evaluated using the CellTiter-Glo luminescent assay kit (Promega, Madison, WI, USA), according to the manufacturer’s instructions. In brief, cells were maintained at a density of 4.8 × 10^3^ per well in 96-well plates. After 24 h, cells were transfected with HPV E6, E6-siRNA1 or E6-siRNA2. Cell viability was measured with CellTiter-Glo reagent according to the manufacturer’s protocols.

CaSki cell apoptosis was analysed using flow cytometry as previously described [77]. Briefly, 60-mm plates were used to maintain 2.6 × 10^5^~3.5 × 10^5^ cells/well. Cells were incubated with FITC-labelled Annexin V and propidium iodide (PI) for 15 min according to the supplier’s recommendation (BD PharMingen, Mississauga, ON, Canada) and detected using a fluorescence activated cell sorting (FACS) Vantage BD FACSCalibur flow cytometer (Becton-Dickinson, Franklin Lakes, NJ, USA).

### 4.3. Construction of Small Interfering RNA (siRNA) and Caspase-3 Activity Assay

The two siRNAs, HPV16 E6-specific siRNA (siE6#1; 5′-GAATGTGTGTACTGCAAGC-3′ or siE6#2; 5′-GCAAAGACATCTGGACAAA-3′) and scramble siRNA, were synthesized by Ambion (Ambion, Austin, TX, USA). Prepared two clones were transfected with oligofectamine (Life Technologies, Gaithersburg, MD, USA) according to the supplier’s recommendation. TRIzol reagent (Life Technologies, Gaithersburg, MD, USA) was used to isolate total RNA, followed by reverse transcription (RT) PCR. The expression of HPV E6 was visualized via an immunoblotting assay. The activity of caspase-3 in CaSki cells was analysed as previously reported [78].

### 4.4. Yeast Two-Hybrid (Y2H) Screening and Quantification of Binding Interaction

The cDNA encoding full-length HPV16 E6 was introduced into the *Eco*RI and *Xho*I restriction enzyme sites of the pGilda/LexA yeast shuttle plasmid, and this complex was used as the bait construct. All screening procedures were conducted as previously reported [79,80]. The β-galactosidase activity was measured using the formula units according to the previously described protocols [79].

### 4.5. Co-Immunoprecipitation (Co-IP) and Western Blot Analysis

The co-immunoprecipitation was performed as reported previously [81]. Briefly, the total cell extracts were incubated with anti-Flag antibody and then precipitated with protein A-agarose [80]. Approximately 20~25 μg of precipitated protein samples were separated by 10~12% SDS-PAGE and electrophoretically blotted onto nitrocellulose membrane (Bio-Rad, Hercules, CA, USA). After blocking, the membranes were probed for HPV16 E6, IRF-1, β-actin, IgG, Bax, Bcl-2, cyclin D1, CDK4, p21, NF-κB, p53, VEGFR-1, VEGFR-2, phospho-VEGFR-2, HIF-1α, VEGF, PI3K, phospho-PI3K, Akt, phospho-Akt, PDK1, phospho-PDK1, mTOR, phospho-mTOR, 4E-BP1, and phospho-4E-BP1, respectively. The protein bands were developed using the ECL chemiluminescence detection kit (GE Healthcare, Piscataway, NJ, USA).

### 4.6. Luciferase, VEGFR-2 Kinase, and PI3K Activity Assays

The HPV16 E6 (HPV16 E6-Luc), p21 (p21-Luc), p53 (p53-Luc), NF-κB (NF-κB-Luc) and VEGFR-2 (VEGFR-2-Luc) luciferase activities were measured as reported previously [82,83]. In vitro VEGFR-2 assays were carried out using the HTScan VEGFR-2 kinase assay kit (Cell Signalling) together with colorimetric ELISA as described previously [81]. The PI3K enzyme activity was assayed as described previously [83,84].

### 4.7. Endothelial Cell Migration and Capillary-Like Tubular Structure Formation

Cell migration analysis was conducted using 8-μm pore-size Transwell (Corning Costar, Cambridge, MA, USA) as previously described [82]. In vitro tube formation was analysed using growth factor-reduced Matrigel (200 μL of 10 mg/mL; BD Biosciences) as reported previously [77,82].

### 4.8. Statistical Analysis

Data are presented as mean ± standard deviations (SD). Statistical comparisons between the different groups were performed using one-way analysis of variance (ANOVA) followed by Student’s *t*-test. A value of *p* < 0.05 was considered to indicate statistically significant difference.

## Abbreviations

HPV	Human papillomavirus
IRF-1	Interferon regulatory factor-1
Rb	Retinoblastoma
E6AP	E6-associated protein
TRADD	Tumour necrosis factor receptor type 1-associated death domain protein
TNFR-1	Tumour necrosis factor receptor 1
FADD	Fas-associated death domain
DISC	Death-inducing signalling complex
LOH	Loss of heterozygosity
AML	Acute myelogenous leukaemia
DBD	DNA-binding domain
NLS	Nuclear location signal
TA	Transactivation domain
VEGFR-2	Vascular endothelial growth factor receptor 2
VEGF	Vascular endothelial growth factor
HIF1-α	Hypoxia inducible factor-1α
HUVECs	Human umbilical vein endothelial cells
siRNA	Small interfering RNA
Y2H	Yeast two-hybrid
Co-IP	Co-immunoprecipitation
